# Prevalence and factors associated with polypharmacy and potential drug interactions in adults in Manaus, Amazonas state, Brazil: a cross-sectional population-based study, 2019

**DOI:** 10.1590/S2237-96222022000200003

**Published:** 2022-06-15

**Authors:** Gustavo Magno Baldin Tiguman, Tayanny Margarida Menezes Almeida Biase, Marcus Tolentino Silva, Taís Freire Galvão

**Affiliations:** 1 Universidade Estadual de Campinas, Faculdade de Ciências Farmacêuticas, Campinas, SP, Brazil; 2 Universidade de Sorocaba, Programa de Pós-Graduação em Ciências Farmacêuticas, Sorocaba, SP, Brazil

**Keywords:** Polypharmacy, Drug Interactions, Pharmacoepidemiology, Drug Utilization, Health Surveys, Prevalence

## Abstract

**Objective:**

To assess the prevalence and factors associated with polypharmacy and the presence of potential drug interactions in Manaus, Amazonas state, Brazil, in 2019.

**Methods:**

This was a population-based cross-sectional study conducted with adults aged ≥ 18 years. The presence of drug interactions among people on a polypharmacy regimen (≥ 5 drugs) was investigated on the Micromedex database. Prevalence ratios (PR) with 95% confidence intervals (95%CI) were calculated using Poisson regression model with robust variance, following hierarchical analysis and considering the complex sample design.

**Results:**

Of the 2,321 participants, 2.8% (95%CI 2.1;3.6) were on polypharmacy regimen, of whom, 74.0% presented drug interactions, usually with four or more drug interactions per person (40.4%) and high severity (59.5%). Polypharmacy was higher among older adults (PR = 3.24; 95%CI 1.25;8.42), people with poor health (PR = 2.54; 95%CI 1.14;5.67), previous hospitalization (PR = 1.90; 95%CI 1.09;3.32) and multimorbidity (PR = 3.20; 95%CI 1.53;6.67).

**Conclusion:**

Polypharmacy was more frequent among older adults and people with medical problems, who presented more drug interactions.

**Study Contributions**

**Main results**

The prevalence of polypharmacy among adults living in Manaus was 2.8% in 2019, higher in older adults and in those who had health problems. With regard to people on a polypharmacy regimen, 74.0% had potential drug interactions, most of which were of high severity.

**Implications for services**

Reducing unnecessary polypharmacy would reduce risks associated with drug interactions, which bring harm to people's health and burden the health system. Strategies aimed at reducing unnecessary polypharmacy include medication reconciliation and deprescription.

**Perspectives**

This population-based survey evaluated potential drug interactions, but it did not evaluate those that are clinically confirmed. Future research evaluating proven interactions may provide better evidence of the magnitude of the problem in the general population.

## Introduction

Simultaneous use of multiple medications has increased worldwide, especially among older adults, possibly due to increased life expectancy and the frequency of multimorbidity in the population.^1^ Greater availability of therapeutic options and recommendations for the use of more than one medication by clinical practice guidelines for the prevention and treatment of diseases may also contribute to this phenomenon.^1^

Polypharmacy is commonly defined as concomitant use of five or more medications.^2^ Although prescribing combination of medications for people with multiple health conditions aims to improve their health, polypharmacy can cause drug interactions and adverse reactions, and severely affect it.^3^ The estimated overall prevalence of adverse drug reactions in Primary Health Care (PHC) is 8%, and it is associated with a greater number of medications used concurrently.^4^ Clinical worsening due to polypharmacy is rarely attributed to the therapy itself. However, it is usually attributed to the clinical conditions of the individual undergoing treatment, making it difficult to identify the problems and their causes and, consequently, the recovery of his or her health.^3^

Polypharmacy is associated with higher risks of falls, frailty, hospitalization and deaths, contributing to the increase in health expenditure.^1^ ^,^ ^5^ Concomitant use of multiple medications increases the complexity of therapies, making it difficult for people undergoing treatment to manage medications and their adherence.^5^ The use of substances such as alcohol and tobacco, associated with polypharmacy, increases the risk of drug interactions, causing health damage.^6^

Studies on polypharmacy focus mainly on specific populations, such as older adults and health service users.^7^ Evidence on the prevalence of polypharmacy in the general adult population is still scarce, particularly in contexts of greater social vulnerability, such as the Brazilian Amazon. Evaluation of potential drug interactions in individuals on polypharmacy regimen may contribute to identify risks associated with combination of therapies at the population level.^8^

This study aimed to assess the prevalence of polypharmacy and associated factors in adults living in Manaus, state of Amazonas, between April and June, 2019, and evaluate the frequency of potential drug interactions among people on polypharmacy regimen.

## METHODS

This was a cross-sectional population-based study conducted with adults (≥18 years old) living in Manaus, between April and June, 2019. This study is part of a larger survey aimed to investigate the use of health services and healthcare supplies in the region.^9^

Manaus, the capital of the state of Amazonas, is located in the Northern region of Brazil and had 2,106,322 inhabitants in 2018, accounting for more than half of the state's population.^10^

The study participants were selected using probabilistic sampling, performed in three stages: census (random), household (systematic) and individual (random), stratified by sex and age.^9^ The sample size was calculated as being 2,300 people, based on the prevalence of health service use in the region (primary outcome of the main survey) of 20%,^11^ 95% confidence level, 2% absolute precision and population estimates of 2,106,322 inhabitants.^10^

Trained interviewers gathered the data interviewing participants face-to-face in their homes. Structured questionnaires were pre-configured in SurveyToGo software (Dooblo Ltd, Israel) and registered on electronic devices (Intel TabPhone 710 Pro). The answers were automatically transmitted to the study database via the Internet and stored in the cloud.

The primary outcome was the prevalence of polypharmacy, defined as the concomitant use of five or more medications.

Information on medication use was obtained by asking the following question: *In the last 15 days (or two weeks), have you taken any medications?* If the answer was 'Yes', the names of the medications were recorded as informed by the interviewee. After data collection, the Anatomical Therapeutic Chemical (ATC) classification system of the World Health Organization (WHO)^12^ was used to classify each medication according to its complete ATC code (all levels). Medications whose names were not available or unreadable were categorized as 'uncoded'.

Secondary outcomes included the frequency of potential drug interactions and drug-alcohol and drug-tabacco interactions among those who reported polypharmacy. The presence of potential drug interactions was investigated by searching the Micromedex database, which provides information on medications, including drug interactions based on scientific evidence.^13^ Medications reported by each participant were inserted into this database and, when drug interactions were found, they were compiled according to the classification of severity level: contraindicated (concomitant use of medications is contraindicated); high severity (potentially fatal or requires medical intervention); moderate severity (may result in clinical worsening or requires a change in pharmacotherapy); or low severity (limited clinical effects). Information quality was categorized as follows: excellent (based on randomized controlled trials); good (there is a lack of well-controlled studies); or regular (pharmacological considerations lead to suspected interaction).^13^

In case of a positive response to alcohol or tobacco dependence among individuals on a polypharmacy regimen, potential drug interactions between medications and alcohol and medications and tobacco smoking were searched on the Micromedex database, being classified according to their severity level (contraindicated; high; moderate; low) and quality of available information (excellent; good; regular).^13^

Independent variables included:

- sex (male; female);
- age group (in years: 18 to 24; 25 to 34; 35 to 44; 45 to 59; ≥ 60);
- economic classification, based on the head of the family schooling, availability of comfort items and urbanization of the surroundings of the household^14^ (A/B, C or D/E, where A represents the wealthiest people and E is the poorest);
- schooling (complete higher education or more; complete high school; complete elementary education; below elementary education);
- marital status (without a partner; with a partner);
- health insurance (no; yes);
- self-perceived health status (good; regular; poor);
- medical consultation in the last 12 months (no; yes);
- hospitalization in the last 12 months (no; yes);
- number of chronic diseases (0; 1; ≥ 2);
- tobacco dependence, based on the validated Brazilian version of the Heaviness of Smoking Index, adopting the cutoff point ≥ 2^15^ (no; yes); and
- hazardous alcohol consumption, measured by the validated Brazilian version of the Fast Alcohol Screening Test, with cutoff point ≥ 3 (no; yes).^16^

The medications reported were optionally confirmed by means of a photographic record of medical prescriptions or medication packaging, if available at home. A pilot study was conducted with 150 participants to assess their understanding of the questionnaire; they were included in the final study sample, and no further corrections were required. Twenty percent of the interviews were audited by telephone to confirm the validity of the data. The interviews were sound recorded and georeferenced by the electronic device used for data collection.

Descriptive statistics were used to calculate the absolute and relative frequencies of polypharmacy in the adult and older adult population, with 95% confidence intervals (95%CI), and to characterize potential drug interactions in participants on a polypharmacy regimen. The differences between the variable categories were analyzed using Pearson's chi-square test. The most commonly used medications among participants on a polypharmacy regimen were described according to their ATC classification.

The prevalence ratios (PR) of polypharmacy with 95%CI by independent variables were estimated by means of Poisson regression with robust variance. A hierarchical model of polypharmacy was built, in which the independent variables were organized at proximal and distal levels, to avoid underestimating the effects of distal variables.^17^ The first level (demographic variables) included the variables 'sex' and 'age group'; the second (socioeconomic variables) included the variables 'economic classification', 'schooling', 'marital status' and 'health insurance'; and the third level (clinical variables), 'health status', 'medical consultation', 'hospitalization', 'number of chronic diseases', 'tobacco dependence' and 'harmful alcohol consumption'.

The variables associated with polypharmacy, with a significance level of p-value < 0.20 at their hierarchical level, were included in the subsequent hierarchical levels. Thus, the variables were adjusted for the covariates belonging to the same original level and for the significant variables of previous levels. Associations with p-value < 0.05 in the adjusted analysis were considered statistically significant. The Wald test was used to assess the significance of variables with multiple categories. Stata 14.2 was used to perform the analyses, considering the sample’s complex design (svy command).

The study project was approved by the Research Ethics Committee of the Universidade Federal do Amazonas through the approval letter No. 3,102,942, issued on December 28, 2018 (Certificate of Submission for Ethical Appraisal No. 04728918.0.0000.5020). All participants signed a free and informed consent form before the interviews.

## Results

**Figure 1:**
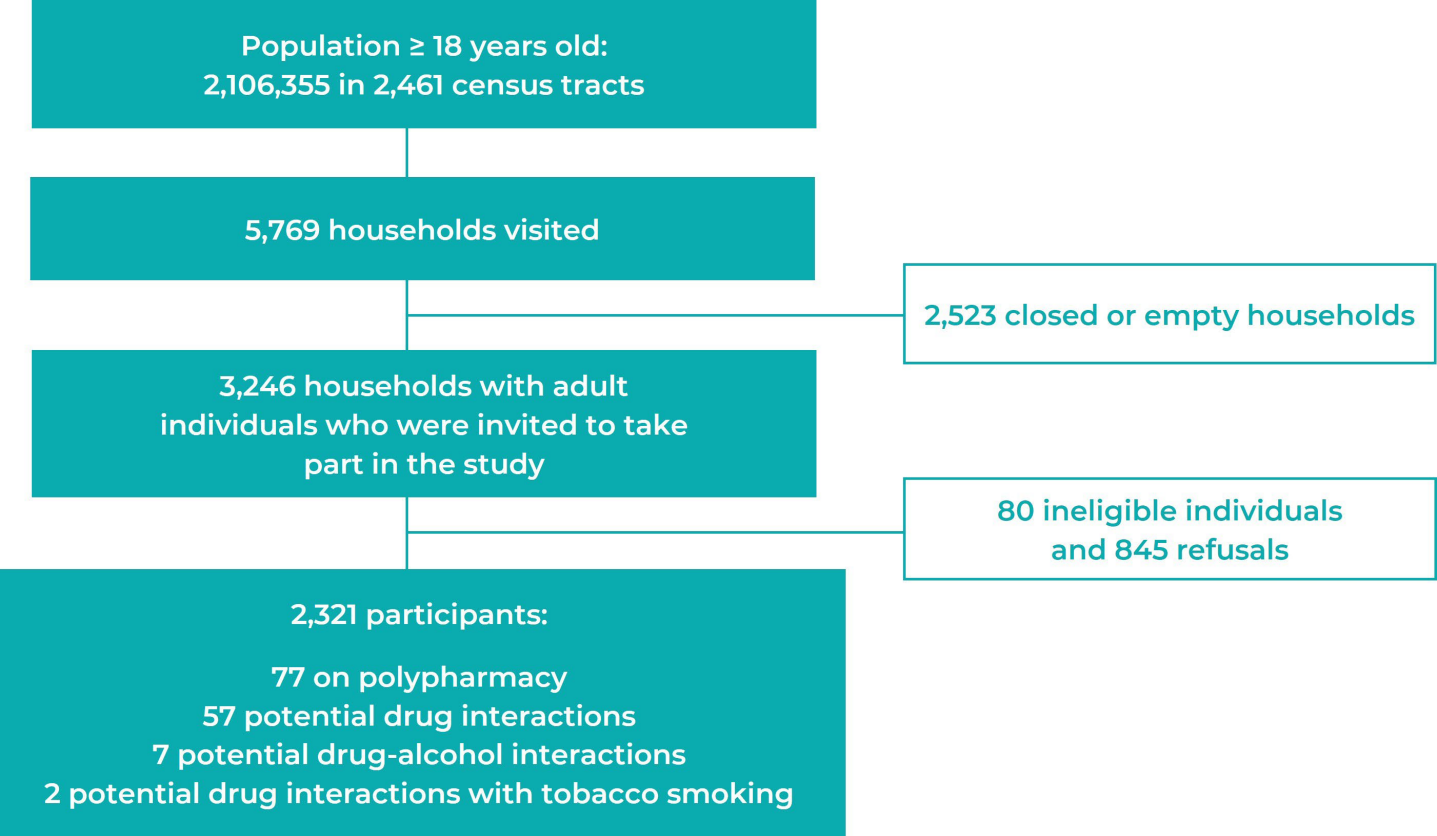
Recruitment process of research participants (n = 2,321), Manaus, Amazonas state, Brazil, 2019

Of the 3,246 households with selected adults who were invited to take part in the study, 80 did not have eligible individuals and 845 refused to participate. A total of 2,321 individuals (Figure 1) were included in the study, of whom 251 (6.7%) were ≥ 60 years. The majority of the participants did not have a partner (62.9%), did not have health insurance (85.5%), reported good health status (67.2%), had consulted a doctor in the last year (73.9%), had not been hospitalized in the last 12 months (89.1%) and had chronic diseases (57.1%) (Table 1).

The prevalence of polypharmacy in the population studied was 2.8% (95%CI 2.1;3.6%), higher in females (3.6%) than in males (2.1%; p-value = 0.010), in older adults (9.0%) compared to younger people (2.7%; p-value < 0.001), and in those with lower education (4.6%) compared to those with complete higher education or more (3.5%; p-value = 0.003). The prevalence of polypharmacy was higher among people who reported poor health status (8.7%) compared to those with good health status (1.5%; p-value < 0.001), individuals who had consulted a doctor in the last 12 months (3.6%) compared to those who had not consulted (0.6%; p-value < 0.001), those who had been previously hospitalized (6.0%) compared to those who had not been (2.5%; p-value < 0.001), and people with multimorbidity (7.3%) compared to those who reported not having chronic diseases (1.1%; p-value < 0.001) (Table 1).

**Table 1:** Description of participants (n = 2,321) and prevalence of polypharmacy in adults (≥ 18 years) and older adults (≥ 60 years), Manaus, Amazonas state, Brazil, 2019

Variables	Adults (n = 2,321)					Older adults (n = 251)				
	Total		Polypharmacy		p-value^a^	Total		Polypharmacy		p-value^a^
	n	%	n	%		n	%	n	%	
**Sex**					0.010					0.110
Male	1,088	51.0	25	2.1		111	49.0	4	9.1	
Female	1,233	49.0	52	3.6		140	51.0	12	8.8	
**Age groups (years)**					< 0.001					-
18-24	405	20.3	12	2.7		-	-	-	-	
25-34	586	31.9	10	1.4		-	-	-	-	
35-44	553	22.2	11	1.6		-	-	-	-	
45-59	526	18.9	28	4.8		-	-	-	-	
≥ 60	251	6.7	16	9.0		251	100.0	16	8.9	
**Economic classification**					0.256					0.900
A/B	282	13.4	13	4.6		21	7.7	1	3.6	
C	1,244	53.7	35	2.5		132	54.2	8	11.6	
D/E	795	32.9	29	2.6		98	38.1	7	6.3	
**Schooling**					0.003					0.425
Complete higher education or more	153	6.9	6	3.5		11	4.3	2	25.9	
Complete high school	1,171	52.5	28	2.2		61	27.3	4	12.9	
Complete elementary education	432	20.4	11	2.6		32	12.0	2	5.3	
Below elementary education	565	20.2	32	4.6		147	56.4	8	6.3	
**Marital status**					0.445					0.127
Without a partner	1,423	62.9	44	2.4		169	64.3	8	3.3	
With a partner	898	37.1	33	3.6		82	35.7	8	19.1	
**Health insurance**					0.597					0.899
No	1,978	85.5	64	2.6		217	85.7	14	6.9	
Yes	343	14.5	13	4.1		34	14.3	2	21.2	
**Health statu**s					< 0.001					0.012
Good	1,498	67.2	22	1.5		112	47.4	3	6.9	
Regular	671	26,8	37	5,0		111	41.3	8	8.5	
Poor	152	6.0	18	8.7		28	11.3	5	19.3	
**Medical consultation^b^**					< 0.001					0.148
No	587	26.1	3	0.6		51	18.7	1	1.4	
Yes	1,734	73.9	74	3.6		200	81.3	15	10.7	
**Hospitalization^b^**					< 0.001					0.393
No	2,071	89.1	58	2.5		233	92.2	14	8.3	
Yes	250	10.9	19	6.0		18	7.8	2	16.1	
**Number of chronic diseases**					< 0.001					0.004
0	921	42.9	9	1.1		43	16.8	-	0.0	
1	682	29.9	11	1.3		55	22.6	-	0.0	
≥ 2	718	27.2	57	7.3		153	60.6	16	14.8	
**Tobacco dependence**					0.828					0.842
No	2,219	95.5	74	2.9		238	92.8	15	9.4	
Yes	102	4.5	3	2.6		13	7.2	1	3.8	
**Hazardous alcohol consumptio**n					0.082					0.265
No	1,871	79.5	68	2.9		234	92.5	16	9.7	
Yes	450	20.5	9	2.5		17	7.5	-	0.0	
**Total**	2,321	100.0	77	2.8		251	100.0	16	8.9	

a) Pearson’s chi-square test; b) In the last 12 months.

The prevalence of polypharmacy in the elderly population was 8.9% (95%CI 2.8;15.1%), more frequent in people with poor health status (19.3%) compared to those with good health status (6.9%; p-value = 0.012), and those with ≥ 2 chronic diseases (14.8%) compared to those who did not have chronic diseases (p-value = 0.004). Among the participants who used at least one medication (n = 1,276), the prevalence of polypharmacy was 5.3% (95%CI 3.9;6.8) among all adult participants and 12.2% (95%CI 4.0;20.4) among older adults (Table 1).

Overall, the use of 442 drugs was reported by the study population on a polypharmacy regimen (Table 2). Losartan (27/442; 6.1%), dipyrone (24/442; 5.4%), acetylsalicylic acid (20/442; 4.5%), simvastatin (18/442; 4.1%), ibuprofen (15/442; 3.4%) and metformin (15/442; 3.4%) were the most commonly used medications.

**Table 2:** Description of the most commonly used drugs (n = 442 medications; 5^th^ ATC level) and main pharmacological groups (1^st^ ATC level) among adult individuals on a polypharmacy regimen (≥ 5 drugs), Manaus, Amazonas state, Brazil, 2019

Medications	ATC^a^ code	n	%
Losartan	C09CA01	27	6.1
Dipyrone	N02BB02	24	5.4
Acetylsalicylic acid	N02BA51	20	4.5
Simvastatin	C10AA01	18	4.1
Ibuprofen	M01AE01	15	3.4
Metformin	A10BA02	15	3.4
Hydrochlorothiazide	C03AA03	11	2.5
Omeprazole	A02BC01	11	2.5
Enalapril	C09AA02	10	2.3
Atenolol	C07AB03	9	2.0
**Pharmacological group**			
Alimentary tract and metabolism	A	89	20.1
Blood and blood-forming organs	B	27	6.1
Cardiovascular system	C	118	26.7
Genito-urinary system and sex hormones	G	5	1.1
Systemic hormonal preparations	H	7	1.6
Anti-infectives for systemic use	J	17	3.8
Antineoplastic and immunomodulating	L	2	0.5
Musculoskeletal system	M	44	10.0
Nervous system	N	67	15.2
Antiparasitic products, insecticides e repellents	P	4	0.9
Respiratory system	R	15	3.4
Sensory organs	S	5	1.1
Herbal medicines	-	3	0.7
Uncoded	-	39	8.8

a) ATC: Anatomical Therapeutic Chemical Classification.

Potential drug interactions were observed in 57 out of 77 participants on a polypharmacy regimen (74.0%). Of the 131 potential drug interactions identified, the majority presented four or more drug interactions per person (40.4%), high severity (59.5%) and regular information quality (51.9%). Seven potential drug-alcohol interactions and two drug interactions with tobacco smoking were identified among participants on a polypharmacy regimen. Regarding drug-alcohol interactions, five presented high severity and good information quality, and two presented moderate severity and regular information quality. With regard to two drug interactions with tobacco smoking identified, both presented high severity and regular information quality (Table 3).

**Table 3:** Main characteristics of potential drug-drug interactions (n = 131), drug-alcohol interactions (n = 7) and drug interactions with tobacco smoking (n = 2) among adult individuals on a polypharmacy regimen, Manaus, Amazonas state, Brazil, 2019

Variables	n	%
**Drug-drug interactions**		
**Number of interactions per person**		
1	25	19.1
2	20	15.3
3	33	25.2
≥ 4	53	40.4
**Severity**		
High	78	59.5
Moderate	50	38.2
Low	1	0.8
Contraindicated	2	1.5
**Information quality**		
Regular	68	51.9
Good	38	29.0
Excellent	25	19.1
Total	131	100.0
**Drug-alcohol interaction**		
**Number of interactions per person**		
1	3	-
2	4	-
**Severity**		
High	5	-
Moderate	2	-
**Information quality**		
Regular	2	-
Good	5	-
Total	7	-
**Drug interactions with tobacco smoking**		
**Number of interactions per person**		
2	2	-
**Severity**		
High	2	-
**nformation quality**		
Regular	2	-
Total	2	-

**Table 4:** Unadjusted and adjusted prevalence ratios (PR) with 95% confidence intervals (95%CI) of polypharmacy among adults (n = 2,321), Manaus, Amazonas state, Brazil, 2019

Variables	Unadjusted analysis		Adjusted analysis	
	PR (95%CI)	p-value^a^	PR (95%CI)	p-value^a^
**Level 1 - Demographic**				
**Sex**		0.070		0.066
Male	1.00		1.00	
Female	1.74 (0.96;3.15)		1.73 (0.96;3.10)	
**Age group (in years)**		< 0.001		< 0.001
18-24	1.00		1.00	
25-34	0.52 (0.21;1.32)		0.51 (0.20;1.27)	
35-44	0.59 (0.24;1.46)		0.58 (0.23;1.41)	
45-59	1.80 (0.84;3.86)		1.75 (0.83;3.71)	
≥ 60	3.35 (1.31;8.59)		3.24 (1.25;8.42)	
**Level 2 - Socioeconomic**				
**Economic classification**		0.256		0.150
A/B	1.00		1.00	
C	0.55 (0.26;1.16)		0.49 (0.23;1.05)	
D/E	0.57 (0.27;1.20)		0.44 (0.18;1.07)	
**Schooling**		0.091		0.546^b^
Complete higher education or more	1.00		1.00	
Complete high school	0.62 (0.23;1.67)		0.80 (0.30;2.13)	
Complete elementary education	0.72 (0.24;2.20)		1.06 (0.33;3.40)	
Below elementary education	1.31 (0.51;3.38)		1.40 (0.49;3.96)	
**Marital status**		0.134		0.148
Without a partner	1.00		1.00	
With a partner	1.51 (0.88;2.60)		1.48 (0.87;2.51)	
**Health insurance**		0.265		0.323^b^
No	1.00		1.00	
Yes	1.56 (0.72;3.38)		1.47 (0.68;3.19)	
**Level 3 - Health**				
**Health status**		< 0.001		0.076
Good	1.00		1.00	
Regular	3.47 (1.79;6.69)		1.89 (0.91;3.91)	
Poor	6.02 (2.88;12.57)		2.54 (1.14;5.67)	
**Medical consultation^c^**		0.002		0.024
No	1.00		1.00	
Yes	2.45 (1.39;4.33)		1.90 (1.09;3.32)	
**Hospitalization^c^**		0.030		0.113
No	1.00		1.00	
Yes	5.86 (1.19;28.86)		3.28 (0.76;14.20)	
**Number of chronic diseases**		0.002		0.024
0	1.00		1.00	
1	2.45 (1.39;4.33)		1.90 (1.09;3.32)	
≥ 2		< 0.001		< 0.001
**Tobacco dependence**	1.00		1.00	
No	1.14 (0.43;3.04)		0.91 (0.33;2.47)	
Yes	6.50 (2.90;14.58)		3.20 (1.53;6.67)	
**Hazardous alcohol consumption**		0.881		0.574
No	1.00		1.00	
Yes	0.91 (0.25;3.34)		0.66 (0.16;2.79)	

a) Wald test; b) Variables removed from the model to adjust the variables of level 3 (p-value > 0.20); c) In the last 12 months.

The *posthoc* analyses indicated that the statistical power of the sample was > 99%. Following the hierarchical model, the following variables were included for adjustments in their original and subsequent levels: sex and age group (level 1 - demographic variables); economic classification and marital status (level 2 - socioeconomic variables); health status, medical consultation and hospital admissions in the last 12 months, and number of chronic diseases (level 3 - clinical variables). The adjusted analysis indicated that polypharmacy was higher among older adults (PR = 3.24; 95%CI 1.25;8.42), people with poor health status (PR = 2.54; 95%CI 1.14;5.67), individuals who had been hospitalized (PR = 1.90; 95%CI 1.09;3.32) and those with multimorbidity (PR = 3.20; 95%CI 1.53;6.67) (Table 4).

## DISCUSSION

Polypharmacy in Manaus was observed in 3% of adults and this prevalence was three times higher in the elderly population. The majority of the medications used by individuals on polypharmacy regimen were antihypertensive, non-steroidal anti-inflammatory drugs and hypoglycemic drugs. Almost three quarters of the people on polypharmacy showed potential drug interactions, mostly presenting high severity and regular information quality. The analysis of the hierarchical model indicated that polypharmacy was higher among older adults, people who had poor health, individuals who had been previously hospitalized and those with multimorbidity.

Recall bias may have influenced the results, given that participants may have forgotten to report some of the medications, potentially underestimating the prevalence of polypharmacy. We sought to minimize this effect by confirming medical prescriptions or medication packaging, when they were available. Drug interactions investigated in this study were theoretical, and they were not clinically confirmed. Some of these interactions may have resulted in clinical effects that have little relevance to the participants.^18^ The three-stage probabilistic sampling method used in this study increased the sample representativeness. However, selection bias may have occurred, because individuals on polypharmacy with severe health conditions might not have been at home due to their health problems.

The prevalence of polypharmacy in adults reported in this study was lower than that found from a Longitudinal Study of Adult Health (12%), which included 14,523 public servants from higher education and/or research institutions located in the Northeast, South and Southeast regions of Brazil.^19^ Polypharmacy was identified in 9% of Brazilian medicine users, and the lowest prevalence corresponded to the North region, according to the National Survey on Access, Use and Promotion of Rational Use of Medicines 2014-2015 (PNAUM), conducted with a subsample of 8,803 adults registered in PHC.^20^ In addition to discrepancies in the representativeness and the contexts of the studies, difficulties in the use and access to health services and medications in the North region may explain these differences, especially among socially disadvantaged and vulnerable individuals.^21^ ^,^ ^22^ Economically advantaged regions tend to offer greater access to health services, including medications, resulting in a possible increase in the number of prescriptions.^19^

The prevalence of polypharmacy was three times higher among older adults compared to general adults (including the elderly). Polypharmacy is a challenge for the ageing population: its prevalence can reach 90%, depending on the definitions of polypharmacy used and the variability between geographic regions.^1^ Professionals in a multidisciplinary team, such as pharmacists, play an important role in monitoring and improving medication use and management in older adult populations, optimizing pharmacotherapy and reducing unnecessary polypharmacy.^23^ Medication reconciliation, a process of creating an accurate identification of the list of medications used by a patient and their comparison with admission, transfer and discharge, presents as an effective strategy for the management of polypharmacy.^3^ Deprescription is another safe, viable and well-tolerated intervention, often conducted by pharmacists, and may result in important clinical benefits for older individuals on a polypharmacy regimen, including reductions in the use of potentially inappropriate medications and the total number of medications used per person.^24^ Platforms such as Deprescribing.org (https://deprescribing.org) can be used as tools to support deprescription by bringing together scientific publications, evidence-based algorithms, case reports, guides and pamphlets related to the topic.

The majority of the medications taken by individuals on polypharmacy were prescribed for the treatment of chronic diseases, although it could be seen that non-steroidal anti-inflammatory drugs were frequently used. These findings are similar to those reported for the Brazilian population on a polypharmacy regimen, in which medications such as simvastatin, losartan, omeprazole, acetylsalicylic acid and metformin are among the most commonly used.^20^ Another study, conducted with 10,528 adults in the United States who reported having chronic conditions in 2009, found that individuals with cardiometabolic diseases (hypertension, diabetes or heart diseases) were particularly at higher risk of polypharmacy, indicating the need for greater monitoring for potential drug interactions in this group.^25^

Almost three quarters of the participants on polypharmacy presented potential drug interactions; more than half of them were of high severity. Although potential serious drug interactions were not clinically confirmed, they may require medical intervention, or may be even fatal.^13^ A previous population-based study conducted with 2,143 older adults living in the Metropolitan Region of São Paulo in 2000 found that 34% of individuals on polypharmacy (defined as the use of ≥ 6 medications) presented potential drug interactions, most of which were of moderate severity (70%), supported by good evidence quality (65%).^26^ The high number of high-severity drug interactions among individuals on a polypharmacy regimen in Manaus indicates a potential need to strengthen pharmaceutical care and promote the rational use of medications in the region.

Polypharmacy was higher among participants who had poor health, those who had been previously hospitalized, and among those with multimorbidity. An analysis of 9,019 older adults in the general population participating in the PNAUM found a a higher prevalence of polypharmacy in people with poor health status, hospitalizations in the previous year and presence of chronic diseases (mainly diabetes and heart diseases).^27^ The Brazilian Longitudinal Study of Aging, conducted with 9,412 older adults in the country between 2015 and 2016, indicated that polypharmacy was associated with multimorbidity and poor self-rated health, as well as a greater use of health services.^28^ A greater number of health problems and the need for multiple treatments may increase the risk of hospitalizations and reduce health-related quality of life.^8^ With increased life expectancy and associated multimorbidity, the risks of damage resulting from polypharmacy also increase.^1^

There was no association between polypharmacy and tobacco and alcohol dependence. Few drug interactions with tobacco smoking and drug-alcohol interactions were observed among individuals on a polypharmacy regimen. Similarly, there was no association between smoking and polypharmacy in a population-based study in England, conducted with 7,730 participants aged 50 years and older, between 2012 and 2013, although the same study indicated a lower frequency of polypharmacy among alcoholics.^29^ Excessive alcohol consumption was not associated with polypharmacy, while former smokers were more prone to polytherapy, according to a cross-sectional study conducted with 1,705 elderly men living in Sydney, Australia, from 2005 to 2007.^30^ Smokers and alcoholics might have died or might have been absent due to diseases at the time of the interview, causing survival bias to the sample. Another possible explanation would be that the participants abandoned dependence due to health problems, affected by reverse causality.

In conclusion, polypharmacy occurred in approximately 3 out of every 100 adults living in Manaus, and was higher among older adults, people who had poor health status, previous hospitalizations and multimorbidity. Almost three quarters of the individuals on a polypharmacy regimen presented potential drug interactions, most of them of high severity and with regular information quality. Reducing polypharmacy through strategies that rationalize the use of medications, such as medication reconciliation and deprescription, will potentially reduce drug interactions and their consequences, especially in the most fragile groups.
